# The sRNA NsiR4 fine-tunes arginine synthesis in the cyanobacterium *Synechocystis* sp. PCC 6803 by post-transcriptional regulation of PirA

**DOI:** 10.1080/15476286.2022.2082147

**Published:** 2022-06-09

**Authors:** Paul Bolay, Luisa Hemm, Francisco J. Florencio, Wolfgang R. Hess, M. Isabel Muro-Pastor, Stephan Klähn

**Affiliations:** a Department of Solar Materials, Helmholtz Centre for Environmental Research, Leipzig, Germany; b Genetics and Experimental Bioinformatics, Faculty of Biology, University of Freiburg, Freiburg, Germany; c de Sevilla, Instituto de Bioquímica Vegetal Y FotosíntesisCSIC-Universidad, Sevilla, Spain

**Keywords:** Cyanobacteria, nitrogen assimilation, arginine metabolism, RNA regulator, sRNA, posttranscriptional regulation

## Abstract

As the only oxygenic phototrophs among prokaryotes, cyanobacteria employ intricate mechanisms to regulate common metabolic pathways. These mechanisms include small protein inhibitors exerting their function by protein–protein interaction with key metabolic enzymes and regulatory small RNAs (sRNAs). Here we show that the sRNA NsiR4, which is highly expressed under nitrogen limiting conditions, interacts with the mRNA of the recently described small protein PirA in the model strain *Synechocystis* sp. PCC 6803. In particular, NsiR4 targets the *pirA* 5ʹUTR close to the ribosome binding site. Heterologous reporter assays confirmed that this interaction interferes with *pirA* translation. PirA negatively impacts arginine synthesis under ammonium excess by competing with the central carbon/nitrogen regulator P_II_ that binds to and thereby activates the key enzyme of arginine synthesis, N-acetyl-L-glutamate-kinase (NAGK). Consistently, ectopic *nsiR4* expression in *Synechocystis* resulted in lowered PirA accumulation in response to ammonium upshifts, which also affected intracellular arginine pools. As NsiR4 and PirA are inversely regulated by the global nitrogen transcriptional regulator NtcA, this regulatory axis enables fine tuning of arginine synthesis and conveys additional metabolic flexibility under highly fluctuating nitrogen regimes. Pairs of small protein inhibitors and of sRNAs that control the abundance of these enzyme effectors at the post-transcriptional level appear as fundamental building blocks in the regulation of primary metabolism in cyanobacteria.

## Introduction

Nitrogen (N) is an essential component of key biomolecules. Given that combined N is a valuable resource, its energy-intensive uptake and incorporation into metabolism is a highly regulated process in bacteria [1,2]. One of the most thoroughly investigated N assimilatory pathways is the glutamine synthetase – glutamine:2-oxoglutarate-aminotransferase (GS-GOGAT) cycle in enterobacteria and most Gram-negatives (for a review see [3]). In this cycle, GS enzyme activity is fine-tuned by a bicyclic cascade of covalent modifications [4–7] that sensitizes the enzyme for feedback inhibition by a suite of molecules involved in N and energy metabolism [8,9]. Moreover, N assimilation is controlled at transcriptional level by the NtrC-NtrB two component system, which regulates the transcription of genes involved in N assimilation, for example, *glnA* that encodes GS [10,11].

Cyanobacteria are major drivers of the global C and N cycles as they perform oxygenic photosynthesis and many representatives also N_2_ fixation [12–17]. Albeit cyanobacteria possess a GS, which is structurally similar to enterobacterial GS [18], its regulation is fundamentally different. For example, cyanobacterial GS is devoid of covalent modifications [19–21]. Instead, most cyanobacteria tune specific GS activity via protein–protein interaction by means of small inhibitory proteins, so-called inactivating factors (IFs) that are deployed depending on the N status of the cell [22,23]. As the widespread NtrB-NtrC system is absent in cyanobacteria, transcriptional regulation of N assimilatory genes is achieved via the global N regulator NtcA, which can act both as activator (e.g. in case of *glnA*) and repressor (e.g. in case of the genes *gifA/B* which encode two different IFs, IF7 and IF17) [24,25].

The binding affinity of NtcA to the respective promoters and hence, its activity as transcriptional regulator is determined in response to 2-OG metabolite levels via the major signalling protein P_II_, in particular the P_II_-PipX-NtcA regulatory axis [26,27]. In bacteria, 2-OG represents a proxy for monitoring the C/N balance of the cell as it provides the carbon framework required as a substrate for N assimilation and its abundance negatively correlates with N availability [28]. As soon as N limitation occurs, 2-OG levels increase, which leads to a release of PipX from P_II_ and complex formation with NtcA, thus activating it for DNA binding. As a result, NtcA fosters N scavenging by activating transcription of N assimilatory genes and by preventing transcription of genes encoding inhibitory proteins.

Beyond N assimilation, regulation of storage and mobilization is achieved by the interaction of P_II_ with N-acetyl-glutamate-kinase (NAGK), catalysing the rate-limiting step of arginine synthesis [29,30]. Again, 2-OG levels determine the interaction of P_II_ with NAGK. As NAGK is feedback inhibited by arginine, P_II_ interaction with the enzyme, which is only permitted during N sufficiency, relieves NAGK from feedback inhibition, and in turn accelerates arginine synthesis [27].

Recently, additional players involved in regulating cyanobacterial N metabolism have been discovered in *Synechocystis* sp. PCC 6803 (from here: *Synechocystis*). In particular, the role of small proteins as effectors for the activity of key enzymes receives more and more attention (for a review see [31]). One such small protein is PirA (P_II_-interacting regulator of arginine synthesis) which has recently been shown to modulate arginine synthesis via competition with NAGK for P_II_ binding [32]. As binding of NAGK to the P_II_ protein reduces the effect of feedback inhibition, PirA interferes with arginine accumulation and acts as indirect inhibitor of NAGK. Similar to *gifA* and *gifB*, which encode the two known proteinaceous GS inhibitors IF7 and IF17, expression of the *pirA* gene (Gene ID: *ssr0692*) increases with rising N availability due to relief from NtcA-mediated transcriptional repression [24,32]. Moreover, novel RNA-based mechanisms regulating N assimilation have been described in *Synechocystis* and other cyanobacteria [33–35].

Besides proteinaceous regulators, bacteria possess diverse regulatory small RNAs (sRNAs) [36]. Many of these sRNAs are only expressed during specific environmental conditions, thus pointing towards specific biological functions. These sRNAs hold great potential for the discovery of novel regulatory mechanisms and metabolic engineering for biotechnological applications [37,38]. Several conditionally regulated sRNAs have been identified in *Synechocystis* [39]. One of these, the nitrogen stress induced RNA 4 (NsiR4) is strongly up-regulated under N limitation. NsiR4 was found to partially control the synthesis of IF7 via direct interaction with the *gifA* mRNA, thereby also controlling glutamine synthesis [40]. The regulons of bacterial sRNAs typically contain multiple target genes and these targets often reveal a distinct physiological role of the respective sRNA. For instance, the sRNA CrfA of *Caulobacter crescentus*, which accumulates under C starvation, promotes the expression of a multitude of genes involved in adaption to C starvation by preventing target mRNA degradation [41]. In *Pseudomonas aeruginosa* O1, the sRNA NalA (nitrogen assimilation leader A) transcribed directly upstream of the nitrate assimilation *nirBD-PA1779-cobA* operon was shown to be essential for transcription of the nitrate assimilation operon by means of transcriptional antitermination [42]. In *Synechocystis*, the sRNAs PsrR1 and IsaR1 were shown to control sets of genes associated with photosynthesis upon shifts in light intensity [43] or iron availability [44]. In the light of these studies, it is very likely that NsiR4 regulates additional genes of N assimilation beyond *gifA* that are yet to be identified.

Here, we show that the *pirA* gene of *Synechocystis* is post-transcriptionally regulated by the small RNA NsiR4, and thereby clearly interferes with arginine metabolism. This extends the role of NsiR4 as a key regulator of N metabolism in cyanobacteria. NsiR4 coordinates N assimilation, storage and remobilization at the post-transcriptional level. Moreover, the NsiR4-*pirA* pair appears as paradigm for the function of modules consisting of a sRNA and one or several small protein regulators that affect major metabolic enzymes.

## Material and methods

### Strains and growth conditions

*Synechocystis* wild type and derivative strains were grown photoautotrophically at 30°C on Cu^2+^-free BG11 medium [45], supplemented with 1 g l^−1^ NaHCO_3_ (BG11C) and bubbled with 1% (v/v) CO_2_ in air, under continuous illumination (50–70 µmol of photons m^−2^ s^−1^; 4000 K LED lights). Prior to the experiments investigating the impact of altered NsiR4 abundance the cultures were supplemented with 1 µM CuSO_4_. Ammonium treatment was carried out by addition of 10 mM NH_4_Cl and 20 mM N-tris(hydroxymethyl)-methyl-2-aminoethane-sulphonic acid (TES) buffer, pH 7.5. The recombinant *Synechocystis* strains used in this study were generated and described previously [40].

### Verification of sRNA:mRNA interaction

For the verification of sRNA:mRNA interactions *in vivo* a heterologous reporter system and the pXG10-SF plasmid were used as described [46,47]. Here, co-expression of a respective sRNA and an mRNA harbouring the 5ʹUTR of the potential target and an open reading frame encoding a reporter gene allows monitoring of the sRNA:mRNA interaction and its regulatory impact on gene expression. For the reporter assay *E. coli* TOP 10 were transformed with different plasmid combinations that harboured either a *superfolder gfp* gene transcriptionally fused to the 5ʹUTR of putative target mRNAs or respective sRNAs and negative controls (nonsense RNA). The primers used for cloning and the resulting plasmids are given in **Supplementary Tables S1** and **S2**. Briefly, the entire 5ʹUTR of *pirA* containing the predicted NsiR4 interaction sequence and a part of the coding region were amplified from *Synechocystis* gDNA using the primer combinations ssr0692_5_NsiI/ssr0692_3_NheI. Further processing was performed as described [40]. For every plasmid combination, six clones were picked to inoculate a liquid culture grown in LB medium with the corresponding antibiotics at 37°C overnight. A volume of 30 μl of each pre-culture were diluted in 170 μl fresh LB medium and fixed with 22 μl 10% Histofix (Roti-Histofix 10%, Carl Roth GmbH). The GFP fluorescence was measured with an Accuri C6 flow cytometer (BD Biosciences) as described [48].

### Western blot analysis

For the analysis of protein accumulation, crude extracts from 2 ml culture were prepared as previously described [49]. Subsequently, proteins were separated via SDS-PAGE [50] and transmitted to nitrocellulose membranes (Bio-Rad). Antibodies against PirA and the loading control TrxA [51] were diluted 1:5,000 (anti-PirA) or 1:10,000 (anti-TrxA). Blocking, incubation and washing of membranes was performed as described [32].

### Determination of arginine

To monitor arginine levels, cells were cultivated in BG11 with conventional nitrate content until an OD_750_ of 0.8 was achieved. Shortly before and after the addition of 10 mM ammonium chloride cells were harvested by centrifugation and snap-frozen in liquid N_2_ followed by ethanolic extraction. Arginine was quantified via a high-performance liquid chromatography as described [52].

## Results

In a previous study, we performed transcript profiling of *Synechocystis* strains overexpressing the regulatory RNA NsiR4 [40]. This study led to the confirmation of two different target genes, *gifA* and *ssr1528*, both of which showed reduced transcript levels due to the diminished loading with ribosomes caused by high coverage of NsiR4 with its respective binding sites on each mRNA. However, the entire NsiR4 regulon in *Synechocystis* is not known and likely comprises many other genes in addition to *gifA* and *ssr1528*, consistent with recent findings for the NsiR4 homolog in other cyanobacteria. In *Nostoc* sp. PCC 7120 NsiR4 also controls the expression of *glpX* encoding bifunctional sedoheptulose-1,7-bisphosphatase/fructose-1,6-bisphosphatase and *pgk* encoding phosphoglycerate kinase [53]. One gene that also showed decreased transcript levels in response to pulse overexpression of NsiR4 in *Synechocystis* was *ssr0692* [40]. This gene was recently elucidated to encode the small protein PirA, a key regulator of arginine synthesis [32]. While secondary effects could not be excluded, we speculated that the *pirA* gene might represent an additional NsiR4 target, especially considering the differential arginine accumulation kinetics of the NsiR4 overexpression strain [40].

### *NsiR4 interacts with the 5ʹUTR of* pirA *and diminishes translation*

**Figure 1.**
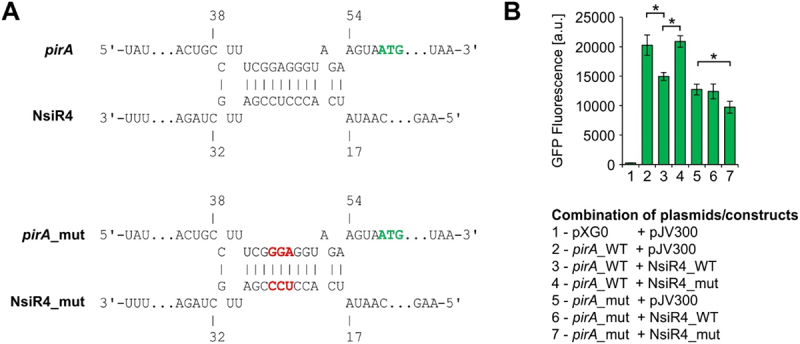
***In vivo* reporter assays for the verification of direct interaction between the 5ʹUTR of *pirA* and NsiR4. A**: Computational interaction prediction between the 5ʹUTR of *pirA* and NsiR4 using the IntaRNA tool [54,55]. The numbers refer to the TSS +1. The start codon of *pirA* is highlighted in green while inserted mutations are highlighted in red. **B**: GFP fluorescence in *E. coli* TOP 10 strains with different plasmid combinations expressing NsiR4 or a nonsense RNA (plasmid pJV300) in presence of the 5’UTR of *pirA* which was fused to a *sfgfp* gene, as well as a negative control accounting for the autofluorescence of the cells (pXG0). It should be noted that the mutation, which was introduced into the *pirA* 5ʹUTR affected translation and hence, the GFP fluorescence detected for these constructs (column 5–6) was generally lower as observed for the WT version. Data are the mean ± SD of two independent experiments each using six independent cultures/clones. Asterisks label statistically different mean values, confirmed by single factor analysis of variance (ANOVA).

To investigate whether NsiR4 bears the potential to bind the *pirA* mRNA, biocomputational RNA–RNA interaction prediction was performed utilizing the intaRNA tool [54,55]. This analysis yielded a complementing region of NsiR4 and the *pirA* 5ʹUTR which stretches from positions −4 to −18 upstream of the *pirA* start codon (see Figure 1a). This arrangement most likely blocks access to the ribosomal binding site by NsiR4 and might interfere with *pirA* translation.

To test whether NsiR4 interacts with the 5ʹUTR of *pirA*, heterologous reporter gene assays developed for RNA–RNA interaction verification in *E. coli* [47] were performed. For that, the 5ʹUTR of *pirA* was fused to a superfolder *gfp* gene and co-expressed with NsiR4. Indeed, co-expression of NsiR4 caused a decrease in GFP fluorescence compared to a negative control omitting NsiR4 expression (Figure 1b). To test whether the observed reduction in GFP fluorescence was solely based on interaction via the predicted RNA–RNA binding region, the respective sequences were mutated in both NsiR4 and the *pirA* 5ʹUTR (see Figure 1a) and different combinations were tested. Mutation of either NsiR4 or the *pirA* 5ʹUTR in combination with the WT version of the respective interaction partner prevented the reduction in fluorescence. However, the reduction in fluorescence was restored as soon as the two mutually compatible versions of mutated NsiR4 and *pirA* 5ʹUTR were combined (Figure 1b). These results unambiguously illustrated that NsiR4 binding to the 5ʹUTR of *pirA* is specific, occurs at the predicted site and interferes with protein synthesis. Given that post-transcriptional regulation of FBP/SBPase and PGK, both key players of the CBB cycle, by NsiR4 was recently demonstrated in the filamentous, diazotrophic strain *Nostoc* sp. PCC 7120 [53] we also tested these interactions in *Synechocystis*. Surprisingly, utilizing the same reporter assays, none of the potential interaction partners of NsiR4 could experimentally be confirmed for any of the variants from *Synechocystis*. Thus, we tested mRNA:sRNA pairs from *Nostoc* sp. PCC 7120 as well. Indeed, we were able to confirm the interaction of this NsiR4 variant with *pgk*, contrasting the situation in *Synechocystis* (**Supplementary Figure S1**).

### *NsiR4 interferes with PirA accumulation in* Synechocystis

**Figure 2.**
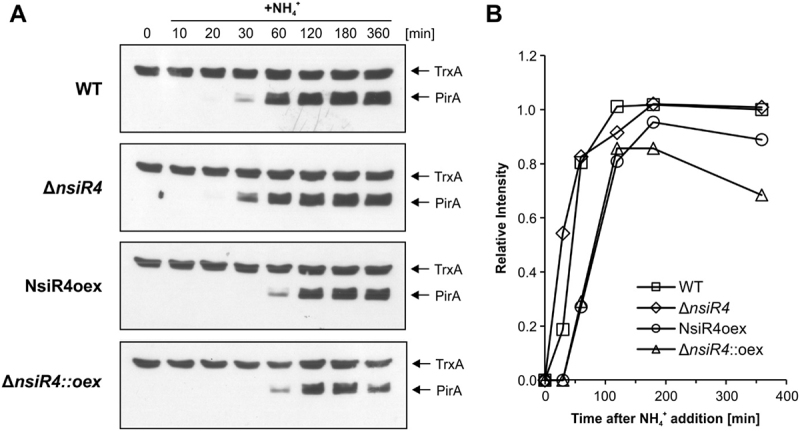
PirA accumulation kinetics in different NsiR4 mutant strains. A: Representative Western blots using antibodies specific against PirA [32]. Equal loading is confirmed by the simultaneous detection of thioredoxin (TrxA) using specific TrxA antibodies. At time point 0, PirA accumulation was stimulated by the addition of 10 mM ammonium (provided as ammonium chloride) to cells that were pre-cultivated in BG11 supplemented with nitrate as sole N source. Prior to sampling all cultures were supplemented with 1 µM CuSO_4_ and further cultivated for 12 hours to ensure sufficient overexpression of NsiR4 in strains NsiR4oex and Δ*nsiR4::oex* as described previously [40]. B: Densitometric analysis of the obtained signal intensity for PirA using ImageJ software. All values were normalized to the corresponding signal for TrxA of the same blot and time point. The data are shown as relative intensities compared to the signal for the WT at 360 min (set as 1).

At this stage, it remained obscure whether the confirmed interference of NsiR4 with *pirA* translation had any relevance in *Synechocystis* as the reporter assays were performed in *E. coli* and a heterologous system might lack factors required to achieve full control. To test if the determined interaction between NsiR4 and the *pirA* mRNA had any effect on the accumulation of the PirA protein in *Synechocystis*, a previously generated PirA-specific antiserum [32] was used. In particular, we investigated three available strains [40] in comparison to the WT, an NsiR4 deletion mutant (Δ*nsiR4*) and two strains ectopically expressing NsiR4 from a plasmid via the Cu^2+^-activated *petE* promoter, either in WT (NsiR4oex) or Δ*nsiR4* background (Δ*nsiR4::oex)*. PirA was described to strongly accumulate in response to ammonium shocks [32]. Interestingly, compared to WT a delayed PirA accumulation in response to ammonium shock was observed for strain NsiR4oex (Figure 2). By contrast, Δ*nsiR4* showed a slightly higher accumulation of PirA 30 minutes after ammonium addition, which was clearly abrogated in strain Δ*nsiR4::oex* (Figure 2). In the light of the reporter assays verifying interaction between NsiR4 and the 5ʹUTR of *pirA*, these results clearly demonstrate that post-transcriptional control by NsiR4 has biological relevance for PirA accumulation kinetics *in vivo*.

### NsiR4-dependent regulation of PirA accumulation affects arginine metabolism

**Figure 3.**
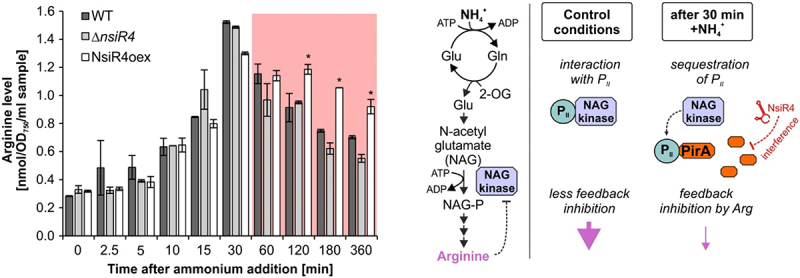
**Implications of enhanced NsiR4 accumulation on arginine metabolism. Left panel**: Shown are arginine accumulation kinetics in WT and strains Δ*nsiR4* and NsiR4oex in response to addition of 10 mM ammonium. Data are the mean ± SD of three independent replicates. Asterisks label significant deviation from the WT controls, confirmed by single factor analysis of variance (ANOVA). **Right panel**: Schematic of the effects of the NsiR4-PirA module on arginine synthesis by modulating NAGK activity before and after ammonium shock.

Our western blots revealed that the regulation of *pirA* by NsiR4 was effective at protein level. Therefore, we set out to determine if this interaction had any relevance for the metabolism of *Synechocystis*. PirA was recently shown to inhibit arginine synthesis by interaction with the regulatory P_II_ protein [32] thereby interfering with NAGK activity. Unfortunately, we were unable to perform reproducible *in vivo* measurement of NAGK activity, thus we monitored arginine accumulation after ammonium shock in the WT and strains Δ*nsiR4* and NsiR4oex (Figure 3). As expected, the internal arginine pool steadily increased in all strains within 30 min. Afterwards, the arginine level began to drop, likely due to the simultaneously accumulated PirA protein that interferes with NAGK activity. This is supported by the fact that a differential accumulation of arginine 60 minutes after ammonium addition was clearly observable for strain NsiR4oex when compared to WT and Δ*nsiR4*, matching the differential PirA protein accumulation determined by western blot (Figure 2). As both WT and Δ*nsiR4* featured a significantly increased PirA content compared to NsiR4oex after 60 minutes, these data nicely illustrate the regulatory impact of NsiR4 on arginine metabolism via post-transcriptional regulation of *pirA*.

## Discussion

The synthesis of arginine is a highly regulated process as this N-rich amino acid represents a central hub for N storage and redistribution in cyanobacteria [56,57]. Beyond its role as a proteinogenic amino acid, arginine serves as building block of N storage polymers, such as cyanophycin [57], polyamines as well as combined N buffer [58]. In cyanobacteria, the process of arginine synthesis is mainly regulated via its rate-limiting step, the reaction catalysed by NAGK which commits N-acetylglutamate towards arginine synthesis via irreversible phosphorylation. This enzyme is feedback-inhibited by arginine and is activated by the master regulator of C/N balancing, the P_II_ protein, in a sophisticated regulatory interplay. This regulation integrates 2-OG levels and ATP/ADP ratios, that is, small-molecule effectors of N and energy metabolism, which are utilized to probe metabolic states of the cell [29,30,59]. Given the vast N and energy requirements of arginine synthesis, it appears that the core principle of its tight regulation in cyanobacteria is to only permit synthesis when sufficient N and energy resources are available (for a review see [58]). Consistently, the recently discovered small protein PirA accumulates in response to high ammonium concentrations and acts as a competitor of NAGK for P_II_ binding in presence of sufficient concentrations of ADP as a measure of low cellular energy status [32].

Interestingly, the ever growing number of characterized RNA regulators has corroborated assumptions that this class of regulators might surpass protein-based regulation in number and diversity [60,61]. Unfortunately, little is known about sRNAs that exert post-transcriptional control on genes involved in arginine metabolism. One of the few examples is the recently characterized 3ʹUTR-derived sRNA *argX* in *Lactobacillus lactis*, which modulates expression of the *arc* operon encoding arginine catabolism genes [62]. The expression of this sRNA is directly regulated by arginine levels and it inhibits the synthesis of arginine diiminase by post-transcriptional regulation, thus directly contributing to control of arginine accumulation. Another example is the dual-function sRNA SR1 of *Bacillus subtilis*, with a multitude of homologs in closely related species, which serves both as regulatory RNA and messenger RNA. In its untranslated state, SR1 inhibits translation initiation of the *ahrC* mRNA, encoding an transcriptional activator of the arginine catabolic operons *rocABC* and *rocDEF* [63,64].

In this study, we show that the cyanobacterial sRNA NsiR4 provides another example as it controls the synthesis of the small protein PirA which in turn interferes with the activity of NAGK. This finding establishes NsiR4 as central regulatory element that coherently executes control on N entry via GS-GOGAT [40] and N stockpiling via NAGK. Interestingly, NsiR4 does not target the corresponding genes directly but controls the expression of two regulatory proteins (IF7 and PirA) that in turn target the activity of these enzymes.

### NsiR4 supports metabolic flexibility during fluctuating N supply via post-transcriptional control of small regulatory proteins targeting amino acid synthesis

**Figure 4.**
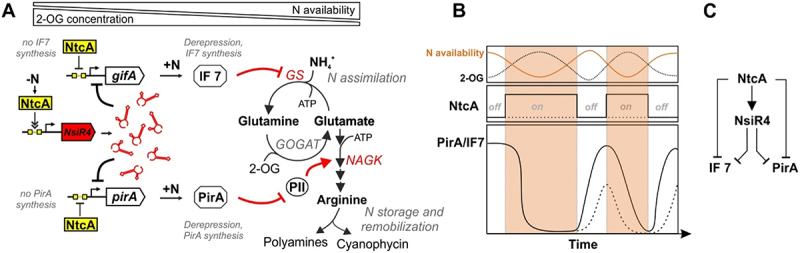
The current status of the regulatory network of NsiR4 in *Synechocystis*. A: Schematic overview of the NtcA-NsiR4-IF7-PirA network and its impact on N metabolism. The network has been built based on data from this study and previous work [25,32,40]. B: Proposed model of the NtcA-NsiR4 feed-forward loop and its effect on the accumulation of corresponding gene products in response to fluctuating N supply (data based on [25,32,40,65,67,72]). The solid line represents target protein expression in a system omitting NsiR4, the dotted line describes a system including NsiR4. C: Regulatory relations between the different players of the NtcA-NsiR4-IF7-PirA network resulting in a multi-output AND-gated type 3 coherent feed-forward loop with a reversed sign-sensitive behaviour [65].

Nitrogen is a vital nutrient and its bioavailable forms are subject to constant fluctuations in many natural environments. When cyanobacteria are exposed to N limitation, 2-OG levels rise and cause a dissociation of P_II_ from NAGK, which enhances the effect of arginine feedback inhibition and in turn decreases enzyme activity [29,30,58]. In *Synechocystis*, NsiR4 expression is strongly induced while both *gifA* and *pirA* genes are repressed by NtcA to promote transition from a resource-conserving to an N scavenging mode (see Figure 4a). NsiR4 interferes with translation of its target mRNAs by base-pairing around the RBS, which interferes with ribosome binding and may also foster degradation of the corresponding transcript. This mode of regulatory interplay, the combination of a transcriptional regulator (NtcA) and an sRNA (NsiR4) which share a target protein (IF7 or PirA) are common to bacterial systems and in that case result in an multi-output AND-gated type 3 coherent feed-forward loop with a reversed sign-sensitive behaviour (see Figure 4c) [65]. This type of regulatory node allows a delayed response of target gene expression to a transcription factor off step, which in this case is the inactivation of NtcA during N excess (see Figure 4b). As cells experience N oversupply, 2-OG levels decline and NtcA becomes inactivated by P_II_-mediated PipX interaction. This results in a de-repression of the *gifA* and *pirA* genes, while the NtcA-mediated activation of NsiR4 is abolished. As a result, residual NsiR4 partially prevents the translation of target mRNA in this transition state, causing delays in the expression of PirA and IF7 during shifts to N-excess conditions. Given that brackish water cyanobacteria such as *Synechocystis* thrive in habitats with strongly fluctuating N supply [66], this mechanism could provide additional metabolic flexibility. Consistently, genomes of the marine genus *Prochlorococcus* which lack NsiR4 [40] appear to also miss homologs of PirA and IF7 [32,67,68]. As these genome-streamlined organisms flourish in nutrient-deficient ultraoligotrophic realms of the ocean, which lack strong fluctuations of nutrient availability, N-cost intensive regulatory systems that fine-tune N assimilation are not required [69–71].

### The NsiR4 regulon beyond cyanobacterial N metabolism

N metabolism is tightly linked to primary C metabolism mainly via 2OG, which is an intermediate of both GS-GOGAT and the tricarboxylic acid (TCA) cycle. Interestingly, based on in silico analyses NsiR4 was predicted to interact with several further mRNAs that encode central enzymes of primary C metabolism, for example, involved in the CBB cycle as well as glycogen synthesis (**Supplementary Table S3**). These included fructose-1,6-/sedoheptulose-1,7-bisphosphatase (FBP/SBPase), phosphoglycerate kinase (PGK), ADP-glucose pyrophosphorylase (AGPase) as well as central components of the photosynthetic electron transport chain that provides reduction equivalents for C metabolism. All these enzymes appear to represent central hubs determining specific routes of C metabolism. By targeting these reactions directly, it could be assumed that NsiR4 might co-determine C flux in response to varying C:N ratios in cyanobacteria. Indeed, in the study by Brenes-Álvarez and colleagues the biological impact of post-transcriptional regulation of the two CBB cycle enzymes FBP/SBPase and PGK by NsiR4 was demonstrated in the filamentous, diazotrophic strain *Nostoc* sp. PCC 7120 representing an additional link between N control and CO_2_ assimilation [53]. Astonishingly, using all variants from *Synechocystis*, none of the potential interaction partners of NsiR4 could experimentally be confirmed using the same reporter assay. Therefore, we also tested the mRNA:sRNA pairs from *Nostoc* sp. PCC 7120. Indeed, interaction of this NsiR4 variant with *pgk* could be confirmed, which is in contrast to the situation in *Synechocystis* (**Supplementary Figure S1**). These results indicate that NsiR4 may need additional factors to interact with its targets in *Synechocystis* or has different target sets in the two strains.

## Supplementary Material

Supplemental Material

## Data Availability

The data that support the findings of this study are available from the corresponding authors S.K. and M.I.M.P. upon reasonable request.
