# Supplementary material for: The sRNA NsiR4 fine-tunes arginine synthesis in the cyanobacterium Synechocystis sp. PCC 6803 by post-transcriptional regulation of PirA
Source: RNA Biol. 2022 Jun 9;19(1):811–8. doi: 10.1080/15476286.2022.2082147 (PMC9196836; doi:10.1080/15476286.2022.2082147)
Supplement: Supplemental Material [file KRNB_A_2082147_SM2539.docx]

# Supplementary Material

Supplementary Table S1: Primers used in this study.

| **Name** | **5’-3’ sequence** | **Used for** |
| --- | --- | --- |
| ssr0692_5_NsiI | AGTTCAATGCATAAATCTAAATGTAAGTTTTTCACTGCCTTTC | Amplification of *pirA* 5’UTR from *Synechocystis* |
| ssr0692_3_NheI | AGTTCAGCTAGCACGCAGGGTTTCTTTGTGGGC |  |
| NsiR4_5_mut23-25 | CACCTCCCGATTGCTAGAGGTCG | Reamplification of plasmid pZE12-luc-  NsiR4 for NsiR4 mutagenesis, mutated nucleotides are underlined |
| NsiR4_3_mut23-25 | TCGGGAGGTGATATTGACTTTATGTCTTGTG |  |
| ssr0692_5_mut | TTCGGGAGGTAGAAGTAATGAATAATCGTAAAC | Reamplification of plasmid pXG10-SF-ssr0692 for *pirA* mutagenesis, mutated nucleotides are underlined |
| ssr0692_3_mut | ACCTCCCGAAAGGCAGTGAAAAACTTAC |  |

Supplementary Table S2: Plasmids used in this study.

| **Plasmid** | **Marker** | **Description** |
| --- | --- | --- |
| pZE12-luc | Ampicillin | General expression plasmid |
| pJV300 | Ampicillin | Control plasmid encoding a nonsense RNA (~ 50 nt) |
| pXG0 | Chloramphenicol | Control plasmid expressing luciferase, used as negative control to measure the autofluorescence of the cells |
| pXG10-SF | Chloramphenicol | Plasmid for the fusion of 5'UTR upstream of sfGFP |
| pXG10-SF-ssr0692 | Chloramphenicol | pXG10-SF plasmid with the 5'UTR and part of *pirA* (*ssr0692)* coding region |
| pZE12-luc-  NsiR4 | Ampicillin | Plasmid expressing NsiR4 |
| pXG10-SFssr0692_  mut | Chloramphenicol | pXG10-SF plasmid with the 5'UTR containing a mutated form of *ssr0692* |
| pZE12-luc-  NsiR4_mut23-25 | Ampicillin | Plasmid expressing NsiR4 with a compensatory mutation to the mutation in *ssr0692_mut* |

**Supplementary Table S3: List of the top 10 predicted interaction partners for NsiR4.** The prediction is based on the CopraRNA algorithm [48,73] using default parameters and NsiR4 sequences from the following strains: *Synechocystis* sp. PCC 6803, *Nostoc* sp. PCC 7120, *Stanieria cyanosphaera* PCC 7437, *Leptolyngbya* sp. PCC 7376, *Rivularia* sp. PCC 7116, *Microcoleus* sp. PCC 7113, *Calothrix* sp. PCC 6303. The CopraRNA algorithm integrates the calculation of hybridization energies for potential RNA-RNA interactions with the respective position related to regulatory elements on the mRNA strand as well as comparative genomics. NA, not available.

| **Rank** | **CopraRNA**  **p-value** | **Locus Tag** | **Gene Name** | **Energy [kcal/mol]** | **Position mRNA** | **Position sRNA** | **Annotation** |
| --- | --- | --- | --- | --- | --- | --- | --- |
| **1** | 1.679e-05 | [*slr2094*](http://www.ncbi.nlm.nih.gov/gene/?term=954653) | *glpX, fbpI* | -12.58 | 79 -- 91 | 21 -- 33 | fructose-1,6-/sedoheptulose-1,7-bisphosphatase I (FBPase/SBPase) |
| **2** | 9.639e-05 | [*slr0394*](http://www.ncbi.nlm.nih.gov/gene/?term=953142) | *pgk* | -7.66 | 108 -- 115 | 26 -- 33 | phosphoglycerate kinase (PGK) |
| **3** | 0.0002729 | [*slr1643*](http://www.ncbi.nlm.nih.gov/gene/?term=952929) | *petH* | -6.81 | 89 -- 95 | 27 -- 33 | ferredoxin-NADP oxidoreductase (FNR) |
| **4** | 0.0006917 | [*sll0109*](http://www.ncbi.nlm.nih.gov/gene/?term=952274) | *aroH* | -12.96 | 76 -- 98 | 18 -- 35 | chorismate mutase |
| **5** | 0.0009146 | [*sll1960*](http://www.ncbi.nlm.nih.gov/gene/?term=954515) | NA | -14.71 | 86 -- 96 | 16 -- 26 | hypothetical protein |
| **6** | 0.001295 | [*slr1039*](http://www.ncbi.nlm.nih.gov/gene/?term=953802) | NA | -15.34 | 18 -- 32 | 18 -- 32 | hypothetical protein |
| **7** | 0.001596 | [*sll1491*](http://www.ncbi.nlm.nih.gov/gene/?term=952007) | NA | -3.74 | 36 -- 42 | 7 -- 13 | beta transducin-like-protein |
| **8** | 0.001983 | [*ssl2009*](http://www.ncbi.nlm.nih.gov/gene/?term=953386) | NA | -8.80 | 8 -- 32 | 11 -- 33 | hypothetical protein |
| **9** | 0.002139 | [*slr1176*](http://www.ncbi.nlm.nih.gov/gene/?term=951909) | *glgC* | -12.00 | 110 -- 144 | 1 -- 34 | glucose-1-phosphate adenylyltransferase. ADP-glucose pyrophosphorylase (AGPase) |
| **10** | 0.002475 | [*slr0951*](http://www.ncbi.nlm.nih.gov/gene/?term=952969) | *ispD* | -8.98 | 131 -- 177 | 1 -- 33 | 2-C-methyl-D-erythritol 4-phosphate cytidylyltransferase |


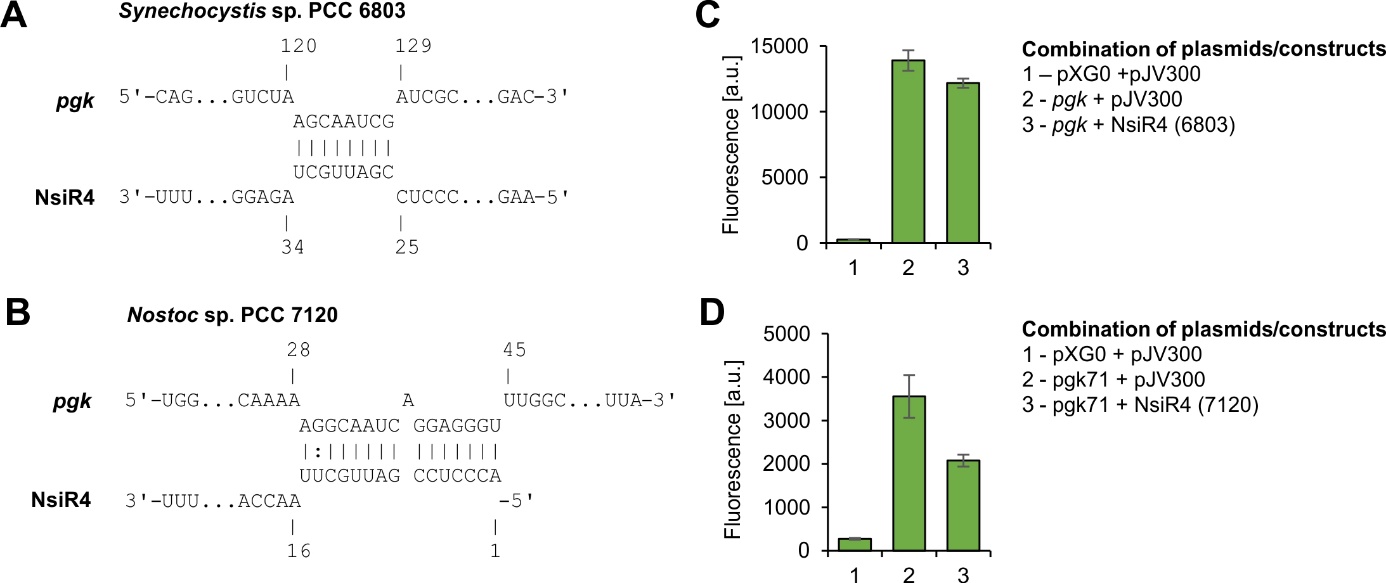


**Supplementary Figure S1: *In vivo* reporter assays for the verification of RNA-RNA interaction.** Here the approach of [47] developed for the verification of RNA-RNA interaction in *E. coli* was followed. Accordingly, the potential target sequence, i.e. the 5’UTR of the respective gene was fused to a superfolder *gfp* gene and co-expressed with NsiR4. Shown are representative data only for the *pgk* gene using the corresponding sequences either from *Synechocystis* (C) or *Nostoc* (D). **A, B:** Computational interaction prediction between NsiR4 and the 5’UTR of *pgk* using the IntaRNA tool [55]. The numbers refer to the respective TSS (+1). **C, D:** Corresponding GFP fluorescence in *E. coli* TOP 10 strains with different plasmid combinations expressing NsiR4 or a nonsense RNA (plasmid pJV300) in presence of the *pgk* 5‘UTRs fused to a *sfgfp*, as well as a negative control accounting for the autofluorescence of the cells (pXG0).
